# Mortality of a large wide-ranging mammal largely caused by anthropogenic activities

**DOI:** 10.1038/s41598-020-65290-9

**Published:** 2020-05-22

**Authors:** M. G. Gantchoff, J. E. Hill, K. F. Kellner, N. L. Fowler, T. R. Petroelje, L. Conlee, D. E. Beyer, J. L. Belant

**Affiliations:** 1 Global Wildlife Conservation Center, College of Environmental Science and Forestry, State University of New York, Syracuse, NY 13210 USA; 2 Missouri Department of Conservation, 65201 Columbia, MO USA; 3 Wildlife Division, Michigan Department of Natural Resources, Marquette, MI 49855 USA

**Keywords:** Ecology, Conservation biology, Ecological modelling, Population dynamics

## Abstract

With efforts to restore large mammal populations following extirpations, it is vital to quantify how they are impacted by human activities and gain insights into population dynamics in relation to conservation goals. Our objective was to characterize cause-specific mortality of black bears (*Ursus americanus*) throughout their range. We first quantified cause-specific mortality for 247 black bears in one harvested and two non-harvested populations. We then simulated a small recolonizing population with and without anthropogenic mortality. Lastly, we conducted a meta-analysis of all published black bear mortality studies throughout North America (31 studies of 2630 bears). We found anthropogenic mortality was greater than natural mortality, non-harvest anthropogenic mortality (e.g. poaching, defense of property, etc.) was greater in non-harvested populations, and harvesting was one of the major causes of mortality for bears throughout their range. Our simulation indicated that removing anthropogenic mortality increased population size by an average of 23% in 15 years. We demonstrated that bears are exposed to high levels of anthropogenic mortality, and the potential for human activities to slow population growth in expanding populations. Management and conservation of wide-ranging mammals will depend on holistic strategies that integrate ecological factors with socio-economic issues to achieve successful conservation and coexistence.

## Introduction

Mortality is a fundamental principle of ecology^1^, however, information on the cause and magnitude of different mortality sources is essential for quantifying the dynamics of wildlife populations. The timing and magnitude of these mortality sources can indicate patterns regarding the ecology, evolution, and conservation issues of different populations^2^. Human activities, such as habitat loss, hunting, and vehicle collisions, are responsible for more than one quarter of global terrestrial vertebrate mortality^3^. Compared to natural mortality, anthropogenic mortality can differ in its impacts across wildlife sex-age classes, and thus have different effects on population demography. For example, mortality from vehicle collisions can alter sex ratios of wildlife populations by disproportionately affecting one sex^4^. Hunting, on the other hand, can remove different individuals than natural predators^5^, modify movement behavior^6^, and influence spatial organization^7^.

Although human activities influence global vertebrate mortality, not all species are equally impacted. Among mammals, larger species are particularly prone to human-induced mortality^3^, and compared to smaller species, anthropogenic mortality increases at a greater rate when landscapes are subject to human activities^8^. Large mammals can experience demographic changes due to human-caused mortality, including direct decreases in numbers and alterations to the sex-age structure of populations^9,10^. In particular, large carnivores have a higher chance to be killed due to a perceived or real threat to human life or property^2^, which can sometimes result in retaliatory killings^11^. From an evolutionary perspective, large-bodied animals are rarely adapted to high adult mortality rates^12,13^, however, the long-term impacts are uncertain because it is difficult to differentiate between evolutionary changes and phenotypic plasticity^14^.

One the largest-bodied mammal groups in North America are bears (Family: Ursidae)^15^. Bears usually have extensive home ranges (e.g.^16,17^), which increases chances of encountering roads and vehicle collisions, as well as the risk of conflict with humans^18,19^. Bears exhibit high flexibility in diet and habitat use that enables them to occupy human-modified landscapes and exploit anthropogenic foods, further increasing the potential for conflict (e.g.^20^). Additionally, bears are valued for their use in traditional medicine^21^ and by hunters^22,23^. Harvesting is the main cause of mortality in hunted bear populations, though it varies across sex and age classes^24^, and although adult female bear mortality is usually lower than for males, it remains a key factor influencing population growth^25,26^.

American black bears (*Ursus americanus*) are the most abundant and spatially expansive ursid in North America, despite having lost almost 40% of their former range due to overexploitation (persecution and unregulated harvest) and habitat loss^27^. Black bears are currently the only large carnivore generally established across the eastern US with stable or increasing populations^28^ and expanding geographic ranges^29^, due to conservation and management plans, as well as repatriation and recolonization efforts (e.g.^30,31^). Since the early 2000s, six states in the US with increasing bear populations have established black bear hunting seasons: Florida, New Jersey and Maryland, after 21-, 33-, and 51-year closures, respectively, and Kentucky, Oklahoma, and Nevada for the first time in their management history^28^.

Effective coexistence with large wide-ranging species, such as bears, hinges on understanding multiple biological, ecological, and socioeconomic factors. With past and current efforts to restore populations of large mammalian species following extirpations (e.g. elk *Cervus elaphus*^32^, wolves *Canis lupus*^33^, black bears^31^, etc.), it is vital to quantify sources of mortality to gain insights into complex population dynamics in relation to conservation goals. Particularly for black bears, hunting is a primary population management tool used by many jurisdictions, but there has been no large-scale assessment of mortality sources across their geographic range. Therefore, our objective was to characterize cause-specific mortality of black bears throughout their range, particularly natural vs. anthropogenic. We hypothesized that: (1) overall mortality of bears would be greater in areas allowing legal harvest, (2) human-caused mortality would be less than natural mortality in areas where hunting was not allowed, and (3) male black bears would represent a greater proportion of overall mortality due to greater mobility and hunter selectivity^34,35^. To achieve this, we first quantified mortality sources for three focal black bear populations: two recolonizing populations for which harvest is not allowed (Mississippi and Missouri) and an established harvested population (Michigan). We then simulated a small recolonizing population to assess the potential for different mortality sources to slow population growth. Finally, we conducted a meta-analysis of all previously published studies of cause-specific black bear mortality to examine the magnitude of natural and anthropogenic mortality on populations of this species throughout North America.

## Methods

### Study areas

Our three focal black bear populations were located in Michigan, Missouri, and Mississippi, USA. In Mississippi, topography is generally flat with elevations from 0 to 247 m above sea level. Vegetation is primarily agricultural land with forested areas along the Mississippi River (Mississippi Automated Resource Information System 2014). Data collection was conducted in the Delta region of western Mississippi, where most black bear sightings occur^30^. Bear density throughout Mississippi was estimated at <1/100 km^2^ (R. Rummell, Mississippi Department of Wildlife, Fisheries, and Parks, pers. comm.). In Missouri, data collection was conducted in the Ozark Highlands where most black bear sightings occur^34^. This region contains karst topography with elevations from about 70 to 280 m and has a humid warm continental climate. Dominant land covers include forest, crop and pasture, grassland, and human developed areas, and black bear density was 1.7/100 km^2^ ^36^. In Michigan, data collection was conducted in the Upper Peninsula. This area has flat topography with elevations ranging approximately from 160 to 240 m, and a humid cold continental climate. Predominant vegetation includes upland and lowland hardwoods, lowland conifer swamps, upland conifers, aspen (*Populus spp*.) stands, row-crop and livestock agriculture, and some herbaceous wetlands^37^. Black bear density is 14–19/100 km^2^ (J. L. Belant unpublished data). There is no black bear harvest in Mississippi or Missouri, while in Michigan they are harvested annually during September and October; only males and females without dependent young are legal to hunt.

### Data collection

Using telemetry to monitor the fate of animals provides less biased estimates than opportunistic encounters of carcasses^38,39^. We used data from long-term black bear GPS-telemetry studies in Michigan (data from 2009 to 2011 and 2013 to 2015, e.g.^37^), Missouri (data from 2010 to 2018, e.g.^40^), and Mississippi (data from 2008 to 2017; e.g.^17^), USA. Collared black bears were monitored using ground and aerial telemetry and were captured or recaptured during summer trapping or winter den checks. In addition, mortality data of collared individuals was obtained through citizen reported events (e.g. harvest, vehicle collisions). A database of all collared bears, including sex and fate, was constructed for the three study areas. Bear fate was defined as the status of the bear at the end of the monitoring period (i.e. alive or dead). We censored bears (i.e., alive at the end of the monitoring period) that either lost their collars in the field, were not re-collared after recapture, or could not be relocated, in which case the last known location was used as the end of the monitoring period. Mortality events were classified as anthropogenic or natural (i.e. human involvement was not documented or confirmed). We further classified anthropogenic mortality into three groups: harvest, non-harvest anthropogenic, and vehicle collision. Non-harvest anthropogenic was defined as any mortality directly caused by humans that excluded harvest (e.g. poaching, legal defense of life or property, etc.). We focused on assessing sub-adult and adult mortality, so only included individuals that were at least 2 years old at the start of the monitoring period.

### Cause-specific mortality analysis

We used a cause-specific mortality risk analysis to compare bear mortality risk among different causes and to assess the effects of multiple covariates on these risks. In a standard Cox proportional hazards modeling framework, it is possible to simultaneously examine the effect of multiple covariates, but the response is the survival time (T) of an individual regardless of the cause. To simultaneously examine the effects of multiple covariates on multiple sources of mortality, we used an extension to the Cox proportional hazards model that jointly considers the survival time and cause of death (K) for an individual bear. We applied the data augmentation approach of^41^ to fit the model, taking advantage of the mutual exclusivity of different risk types^42^. In the data augmentation approach, the dataset of survival times and associated covariates is duplicated Q times where Q is equal to the number of competing risks K. A dummy group variable is created for the augmented dataset and each of the Q “risk sets” are assigned to a unique value of the grouping variable. Then, within each risk set Q, only deaths from the corresponding risk K are included; thus, the sum of deaths in the augmented dataset is equal to the number of deaths in the original dataset^42^.

We fit Cox proportional hazards models to the augmented dataset, and for each model, survival time was modeled as a function of the interaction of the grouping variable (i.e. the type of mortality event) with other covariates of interest (Method A^41^,). Coefficients for the grouping variable represented differences in the degree of risk from different mortality types, and the interactions between the grouping variable and other covariates allow for estimation of how these covariates affect each individual mortality risk^41^. We tested that the assumption of proportional hazards was met for each model using the method of^43^.

Using the approach described above, we fit two Cox proportional hazards models. The first considered two competing risks: anthropogenic mortality (pooling across all types) and natural mortality, and included two covariates: bear harvest status (allowed or not allowed) and sex. The second model considered all types of anthropogenic mortality separately: harvest, non-harvest anthropogenic, vehicle collisions, and natural mortality. A detailed exploration of non-harvest anthropogenic mortality causes was not possible due to low or uneven sample sizes. We included bear harvest status as a covariate in the second model. We set a significance threshold of α = 0.05 for all analyses. Model fitting was conducted with R^44^ using package “survival”^45^.

### Population simulation

We used a simulation approach to assess how anthropogenic mortality might affect bear population growth in a small recolonizing population. We obtained annual survival estimates of male and female adult bear (ages 4+) in the non-harvested areas (Missouri and Mississippi) from our fitted mortality model under two scenarios: natural mortality only, and both natural and anthropogenic mortality (assuming anthropogenic mortality was 100% additive). We input the two annual survival probability scenarios to demetR, a population simulation program that applies a Bayesian projection matrix approach^46^. The program provides average and range estimates for black bear demographic parameters (e.g. litter size, sex ratio, survival of other age classes, etc.) from published literature. We modified the default litter size values (2.0–2.2) to more closely match the observed lower reproductive rate of out two recolonizing populations (1.6–2.2, J. Belant unpublished data). Demographic values for each stochastic population run were drawn from a uniform distribution bounded between values provided. We simulated 100 stochastic populations of 100 individuals (50:50 F:M) over 15 years for each scenario and compared the population trajectories and final population sizes.

### Meta-analysis

We used the database CauseSpec^47^ to obtain studies of adult black bear cause-specific mortality. CauseSpec includes all published studies that used telemetry to monitor terrestrial vertebrates and determine cause of death. For each study, we classified mortalities as direct anthropogenic (harvest, poaching, vehicle collision, etc.) or direct natural (e.g. predation, disease, starvation). Poaching was defined as a bear being killed in an area where harvest was prohibited or outside of a hunting season. We also recorded the number of total mortalities of known cause and the midpoint of the range of years over which the study was conducted. We calculated the proportion of mortality due to each cause (i.e. number dying from the cause/number dying from all known causes).

We used linear models to determine the best set of predictor variables for proportion of mortality due to each mortality source of interest (i.e. overall anthropogenic, harvest, poaching, and vehicle collision). We weighted cases by the number of known-cause mortalities so that studies with a smaller sample size did not have an overly large contribution to models. Since the response variables were proportional data, we performed a logit-transformation^48^. We included if the population was harvested or non-harvested during the duration of the study as a fixed effect, and the midpoint year to account for temporal variation. We ranked all possible model combinations based on sample-size corrected Akaike’s information criterion (AICc) and used multi-model inference to calculate a weighted average of parameter estimates with 95% confidence intervals across competing models^49^.

## Results

### Cause-specific mortality analysis

We collected survival data from 247 black bears across the three study areas (Table 1). Bears were monitored for an average of 24.8 months (SD = 24.5, range = 1–101). Of these, 76 (31%) died with 89% (68/76) due to anthropogenic causes and 11% due to natural causes.

**Table 1:** Number of American black bears in the three focus populations by sex, state, and cause of mortality.

State	Sex	Harvest	Non-harvest anthropogenic	Vehicle collision	Natural	Censored	Total
Michigan	F	17	0	0	1	11	29
Michigan	M	32	0	0	0	10	42
Missouri	F	0	3	0	3	53	59
Missouri	M	2	5	3	2	66	78
Mississippi	F	0	1	0	1	20	22
Mississippi	M	0	2	3	1	11	17
Total		51	11	6	8	171	247

Data from Michigan (2009–2011 and 2013–2015), Missouri (2010–2018), and Mississippi (2008–2017), USA.

**Figure 1:**
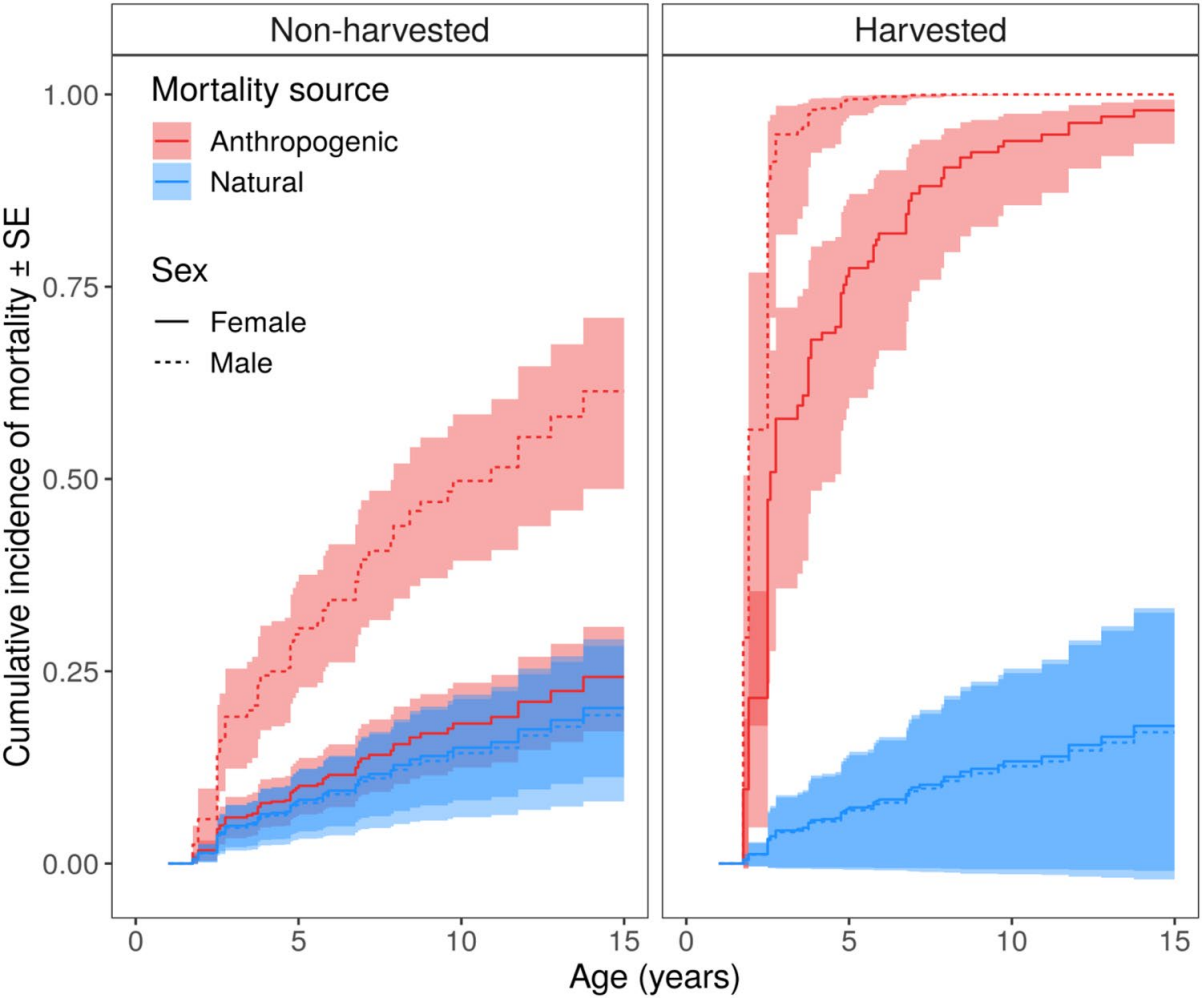
Cumulative incidence functions for black bear mortality cause (anthropogenic or natural) by sex and hunting status: non-harvested (Missouri, Mississippi) and harvested (Michigan).

**Figure 2:**
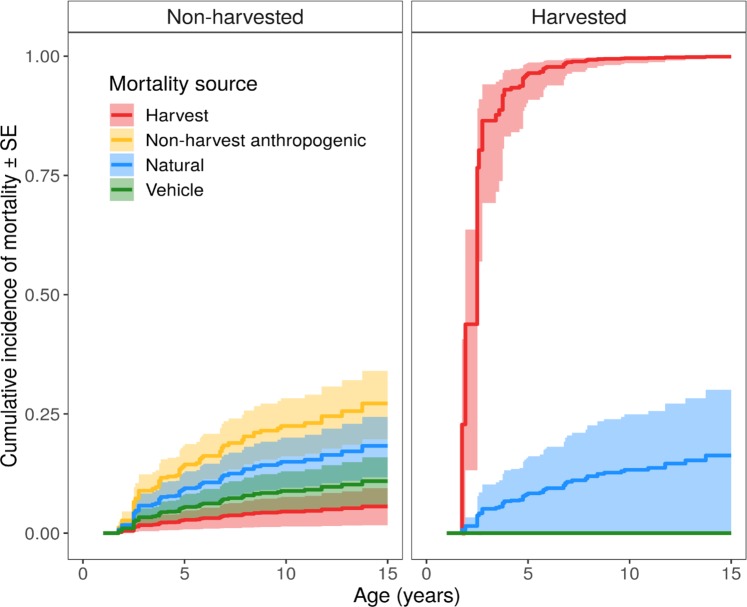
Cumulative incidence functions for more detailed black bear mortality sources for non-harvested (Missouri, Mississippi) and harvested (Michigan) study areas. Two male bears collared in Missouri were hunted legally in Arkansas.

Anthropogenic mortality was the dominant mortality source for all populations (Figs. 1, 2). The relative risk of anthropogenic mortality (derived from the Cox model) was 14 times higher (*P* = 0.01, Table 2) in the study area where harvest was allowed (Michigan) relative to study areas where it was not (Mississippi and Missouri). Across all study areas, we found that the relative risk of anthropogenic mortality among male bears was 3.42 times higher than for females (Table 2). There were no significant differences in natural mortality across study areas, due to harvest status (allowed or not), or by bear sex (Table 2, Fig. 1). Female mortality from anthropogenic and natural causes was similar in the areas with no harvest, but relative risk of anthropogenic mortality was 20 times higher in Michigan (Fig. 1).

**Table 2:** Black bear competing risks model output.

Parameter	Estimate	SE	*P*
Sex [Male]	−0.052	0.736	0.944
Harvest status [Allowed]	−0.135	1.074	0.900
Cause of death [Anthropogenic]	0.208	0.567	0.714
Sex * Anthropogenic	1.283	0.792	0.105
Harvest status * Cause of death	2.772	1.110	0.013

Data from Michigan (2009–2011 and 2013–2015), Missouri (2010–2018), and Mississippi (2008–2017), USA. Model included two mortality causes (anthropogenic and natural) using the Cox regression data augmentation approach of Lunn and McNeil (1995).

In the study areas without bear harvest, mortality was similar for all types of anthropogenic causes (Table 3, Fig. 2). Harvesting mortality in these states would have been zero, except that two bears captured in Missouri were legally harvested in adjacent Arkansas (Table 1). In Michigan, all anthropogenic mortality was due to harvest, which was greater than natural mortality (*P* < 0.01, Tables 1 and 3, Fig. 2).

**Table 3:** Black bear competing risks model output.

Parameter	Estimate	SE	*P*
Harvest status [Allowed]	−0.128	1.070	0.905
Cause of death [Harvest]	−1.254	0.802	0.118
Cause of death [Vehicle]	−0.560	0.627	0.372
Cause of death [Non-harvest anthropogenic]	0.452	0.483	0.350
Hunting status * Harvest	4.900	1.292	<0.001*

Data from Michigan (2009–2011 and 2013–2015), Missouri (2010–2018), and Mississippi (2008–2017), USA. Model included four mortality causes (harvest, vehicle collisions, non-harvest anthropogenic, and natural) using the Cox regression data augmentation approach of Lunn and McNeil (1995).

### Population simulation

**Figure 3:**
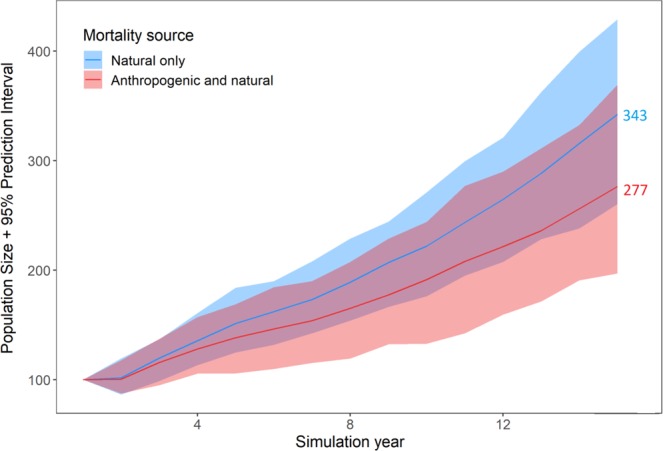
Simulated black bear population trajectories (*n* = 100) over a 15-year period under two scenarios; only natural mortality for adult bears (+4 years old), or both natural and anthropogenic mortality. Colored values indicate average population size at 15 years. Initial population was 100 individuals.

For the natural mortality only scenario, the estimated adult (4+ years) yearly survival probability based on the model was 0.986 (95% confidence interval: 0.973–0.998) for females and 0.986 (0.970–1.00) for males. In the scenario including natural and anthropogenic mortality, yearly female survival probability was 0.968 (0.947–0.991) and yearly male survival probability was 0.928 (0.882–0.974). Population trajectories generated over 15 years showed slower population growth for the scenario that included both mortality sources (Fig. 3). After 15 years, the projected population size for the natural mortality only scenario was 23% higher than for the scenario including anthropogenic and natural mortality (Fig. 3). Over 15 years, the average annual growth (lambda) was 1.071 (1.064–1.085) for both mortality sources combined, and 1.092 (1.072–1.097) after removing anthropogenic mortality.

### Meta-analysis

**Figure 4:**
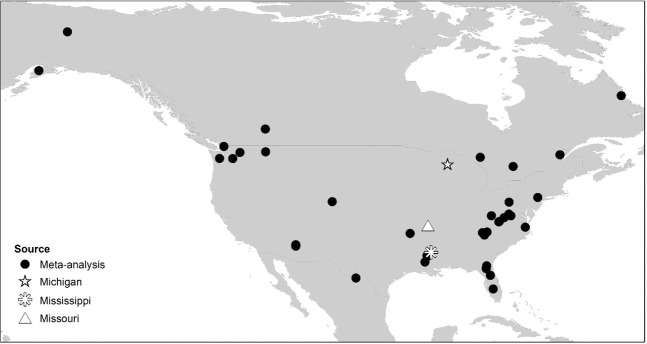
Locations of black bear study sites (n = 31) used in meta-analysis and locations of focus populations in Michigan, Missouri, and Mississippi.

**Figure 5:**
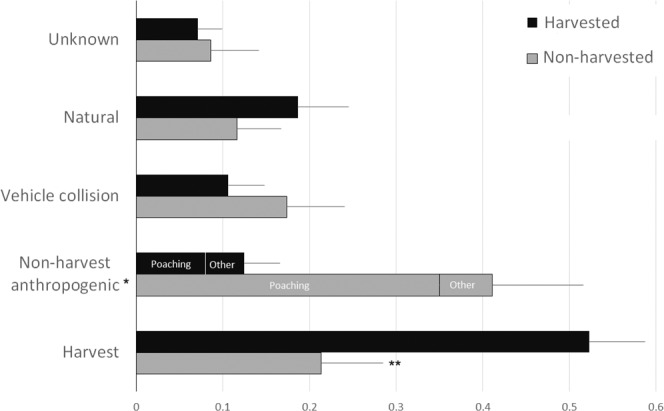
Proportion of cause-specific mortality (± standard error) of black bears monitored by telemetry in North America (n = 536 mortalities from 31 studies) from the meta-analysis. *Non-harvest anthropogenic is any mortality directly caused by humans that excluded harvest. **Harvest mortality in non-harvested populations is a result of individuals moving outside the non-harvest areas where they were collared.

Our dataset for analysis consisted of 31 studies that collectively monitored the fates of 2630 individual adult and sub-adult black bears (Fig. 4). The 31 studies monitored bears across 37 study sites, 12 sites with no legal harvest and 25 with harvest. There were 536 mortalities for which the cause of death was identified. Overall 77% of mortality was due to direct anthropogenic causes (Fig. 5), and anthropogenic mortality was not significantly influenced by the presence of a hunting season (β = −0.123, p = 0.668) or study year (β = −0.013, p = 0.547). As found for the focus populations, harvest mortality also occurred for bears from non-harvested populations. We found, predictably, that harvest was lesser for bears collared in non-harvested areas (β = −1.976, p < 0.001) and did not change over time (β = −0.009, p = 0.678). Mortality from non-harvest anthropogenic origin was greater in non-harvested areas (β = 1.219, p = 0.001) and decreased over time (β = −0.055, p = 0.016). Mortality from vehicle collision was not influenced by harvest status (β = 0.295, p = 0.407) but increased over time (β = 0.059, p = 0.004).

## Discussion

Understanding the degree to which species are impacted by different human activities is key for effective conservation and sustainable management of wildlife populations. Overall, we found that humans are the dominant source of black bear mortality in both the harvested and non-harvested populations that we examined, which spanned a substantial portion of black bear range. For our three focal populations, anthropogenic mortality was greater for males, and both vehicle and non-harvest anthropogenic mortality were more prevalent in non-harvested areas. The results of the meta-analysis suggested similar patterns; anthropogenic mortality overall was twice as prevalent as natural mortality, non-harvest anthropogenic mortality (particularly poaching) was more prevalent in non-harvested populations, and harvesting was one of the major causes of mortality for bears. Results supported our first and third hypotheses (larger mortality for harvested populations, and for males). Our second hypothesis, human mortality being lower than natural mortality for non-harvested populations, was not supported.

Despite being the largest mortality source, anthropogenic mortality appears to be low overall. Since black bears have been extirpated in many areas in the past due to persecution and overharvest, our findings signal conservation success. For other carnivores, such as brown bears (*Ursus arctos*) or pumas (*Puma concolor*), hunting represents over 50% of adult male mortality in some populations^50,51^; though the actual percentage of the total population being harvested can be low (<5%). Similarly for other large mammals, legal harvests and illegal kills were large components of mortality throughout their range (e.g. elk^52^), and hunting and vehicle collisions accounted for nearly half of all mortality of adult medium and large North American mammals^2^.

Hunting is the most easily managed component of mortality in many wild populations as well as the easiest to assess^53^. The meta-analysis revealed that harvest is a large source of anthropogenic black bear mortality, even for bears collared in areas where there is no legal harvest. Larger-bodied species such as black bears are particularly prone to this occurrence due to their wide-ranging movement, which often take them outside non-harvest areas^54^. Selective hunting has the potential to affect population dynamics^9^, and evolutionary processes; though strong evidence of artificial selection has only been found in mountain ungulates^55^. Hunter selectivity appears to be influenced by many factors, such as regulations or social preferences, perceived opportunities, or quota availability^14,53^. Particularly for bears, sex and age-specific vulnerabilities to hunting have been found in many species^50,56–58^ pointing to selectivity for younger, inexperienced individuals, especially males. In many areas, the killing of females with dependent offspring is prohibited or discouraged (e.g.^56,59,60^ which can cause life history alterations^61^. Many wildlife species are managed expecting hunting mortality to trigger density-dependent responses in reproduction, survival, and population growth (compensatory mortality hypothesis^51^). We found that the overall mortality in the harvested focus population was greater than in non-harvested populations, and, supporting this hypothesis, the average litter size was also greater in this population (2.6 in Michigan vs 1.9 in Missouri and 1.8 in Mississippi^62^). However, as bear density is lower in the non-harvest areas (see Methods), a density-dependent effect cannot be excluded. Nonetheless, a trade-off between reproduction and survival has been previously quantified for black bears throughout their range^24^.

Sustainability is a fundamental principle underlying managed wildlife harvests. Particularly for black bears, hunting is a primary population management tool used by many jurisdictions throughout their range. In many managed vertebrate populations, the selective pressures of human activities have raised concern regarding their long-term evolutionary consequences^2,63^; however, robust evidence is lacking^14^. Despite this concern, hunting is an important component of many conservation programs and can provide economic and social incentives to encourage support for wildlife conservation^64,65^, including mitigating the socioeconomic and political costs of the coexistence with wildlife^66,67^. In the USA, a large percentage of funding for wildlife conservation originates from the Federal Aid in Wildlife Restoration Act (which taxes firearms and ammunition), and hunting licenses^68,69^. Overall, the potential role of hunting in conservation, with some conditions, has been recognized by the IUCN^70^.

The meta-analysis revealed that non-harvest anthropogenic was the greatest mortality source for unharvested populations, and the second greatest mortality source for harvested populations. A carnivore non-harvest mortality can occur under many different scenarios, such as poaching (i.e. illegal hunting or trapping in an area or time of year in which harvest is not allowed); a result of perceived or real threat to a person, property, or domestic animals; or retaliatory killing due to perceived economic loses^71^. As large carnivores, bears can threaten people or property, and are sometimes killed to reduce this threat, resulting in population sinks for bears in areas with more dense human populations^72^. However, low reported numbers of non-harvest mortalities does not necessarily infer a correspondingly low number of human-bear conflicts. For example, the presence of a hunting season, especially with liberal hunting regulations (e.g. year-round hunting, baiting allowed), can mask many defense of life or property mortalities because they are reported as sport kills^72^. On the other hand, the illegal killing of carnivores is often poorly quantified and understood due to its secretive nature^73^, yet its effects can be substantial. For example, poaching is the most likely cause of European Lynx (*Lynx lynx*) stagnated population growth^74^ and lack of expansion^75^ in certain populations. To reduce conflict, governments might implement lethal depredation management, which was found to decrease poaching of wolves in Wisconsin, USA^67^. The illegal killing of carnivores is an especially challenging conservation issue in socio-ecological systems^76^ and the relationship between harvest, poaching, and effective carnivore management is complex. For recolonizing black bear populations in North America, increased abundance or density will inevitably result in more conflicts, and a combination of factors such as education and outreach, garbage ordinances, and adverse conditioning, can help decrease non-harvest anthropogenic mortalities^77^ as well as improve public attitudes and tolerance^78^.

Our population simulations indicated that if natural and anthropogenic mortality sources were completely additive, non-harvest anthropogenic mortality would have a considerable effect on a colonizing black bear population in less than 15 years. Even though there was a small decrease in lambda (0.021) and in both scenarios the population increased, when including anthropogenic mortality (non-harvest), the resulting population was 23% smaller. Under alternative scenarios where lambda is closer to 1, such a decrease in lambda could limit population growth or push a stable population toward decline. However, we recognize that anthropogenic mortality is likely neither purely additive nor compensatory and suggest the long term effect would likely be reduced but still present. It is possible that some of the natural mortalities in our focal populations may have been anthropogenic in origin, and that some anthropogenic mortalities were of animals that were sick, starving, etc. pointing to a compensatory effect. For other large North American carnivores, such as wolves or pumas, it is unclear if human-induced mortality is mostly compensatory or additive^42,79,80^. Whether direct anthropogenic mortality can slow recolonization processes or range expansions of carnivores is a difficult question^75^, involving complex processes in which ecological elements act together with human related elements, such as legal protection and public tolerance^11,81–83^. Species-specific responses to human activities, and whether they can slow recolonization or range expansion, will likely be a result of a combination of traits such as reproduction potential, body size, and taxonomic group, as well as the likelihood of conflict and the public’s attitude towards the species^73^. In many areas, the desired management outcome is to slow carnivore population growth and expansion, yet some species are extremely resilient to anthropogenic mortality, such as the coyote (*Canis latrans*), which despite common lethal management^84^ continues to expand throughout North America^85^.

Bear-vehicle collisions appeared to be lesser in the focal harvested population relative to the non-harvested populations, yet we cannot attribute it to a change in behavior without considering the differences among study areas regarding road density and traffic volume. Moreover, we found no differences in collisions between harvested and non-harvested populations in the meta-analysis. All the vehicle collisions in our three focal populations involved male black bears, likely a result of males ranging over larger areas than females^17^ which increases the odds of crossing a road^86^ as well as the typical higher tolerance of male bears to human disturbance^87^.

Our meta-analysis is subject to some biases and methodological constraints. Oftentimes, the populations being monitored by telemetry are of management concern, which tend to be heavily impacted by humans. Such biases in which populations are apt to be monitored could have led to overestimates of anthropogenic mortality as they are not a random sample of all bear populations. We also only considered the proximate mortality cause, which can have different underlying causes for which we do not account. For example, natural factors such as food shortages can cause bears to move into areas dominated by humans where they are more likely to suffer anthropogenic mortality^88^. Finally, we ignored mortalities of unknown cause, which may be biased toward certain mortality sources such as poaching^89^.

## Conclusions

Quantifying and understanding the relationships between anthropogenic and natural mortality is essential for planning sustainable conservation of wildlife^90^. We demonstrated that black bears are exposed to high levels of anthropogenic mortality in large portions of their range, and how harvest can influence other types of anthropogenic mortality. Even in partially recovered and sustainably managed carnivore populations, regulated hunting can still influence their ecology^61,91^. On the other hand, poaching is often lower within protected areas (e.g. national parks^92^), but these areas are often insufficient to support viable populations of wide-ranging species^93,94^. Mammalian species are increasingly challenged by new mortality causes as human development expands into their habitats^2^, and although carnivores can adapt to humans, there remains a strong need to understand and integrate their management into non-protected landscapes^95^. Effective coexistence with large and wide-ranging vertebrates, particularly species with expanding ranges, will depend on holistic strategies that integrate ecological factors, such as habitat protection and landscape connectivity, with socio-economic factors, such as harvest management, public opinion, and conflict mitigation.

## Data Availability

Data used for the meta-analysis is freely available at CauseSpec: 10.1002/ecy.2865.
